# Circular RNA ZNF609 promotes laryngeal squamous cell carcinoma progression by upregulating epidermal growth factor receptor via sponging microRNA-134-5p

**DOI:** 10.1080/21655979.2022.2034703

**Published:** 2022-03-02

**Authors:** Xiaoyan Yin, Jingmiao Wang, Chunguang Shan, Qiaojing Jia, Yanrui Bian, Haizhong Zhang

**Affiliations:** Department of Otolaryngology, Head & Neck Surgery, The Second Hospital of Hebei Medical University, PR. China

**Keywords:** CircRNAs, ZNF609, miR-134-5p, EGFR, laryngeal squamous cell carcinoma

## Abstract

Emerging evidence has revealed that aberrantly expressed circular RNAs (circRNAs) play vital roles in tumorigenesis and progression of diverse human malignancies. CircZNF609 was found to be involved in hepatocellular carcinoma, but the role and underlying mechanism of circZNF609 in laryngeal squamous cell carcinoma (LSCC) remain unclear. This study aimed to explore the molecular mechanism of circZNF609 in LSCC. qRT-qPCR was performed to detect the expression of circZNF609 and microRNA-134-5p (miR-134-5p) in LSCC. Colony formation assay, CCK-8 assay, BrdU incorporation assay, clone formation assay, transwell invasion assay and Western blot analysis were performed to evaluate LSCC cell proliferation, as well as the expression of proliferating cell nuclear antigen (PCNA) and MMP-2. Luciferase reporter assay, target gene prediction and screening were used to validate downstream target genes of circZNF609 and miR-134-5p. EGFR expression was detected by Western blot analysis and RT-qPCR. Nude mice were used to detect tumor changes. CircZNF609 was upregulated in LSCC and associated with poor survival of LSCC patients. Knockdown of circZNF609 inhibited LSCC proliferation, invasion and the expression of PCNA and matrix matalloproteinases-2 (MMP-2). CircZNF609 can regulate miR-134-5p to upregulate epidermal growth factor receptor (EGFR). In addition, knockdown of EGFR or overexpression of miR-134-5p could reverse the tumor-promoting effects of circZNF609 in LSCC. In LSCC tissues, circZNF609 was negatively correlated with miR-134-5p and positively correlated with EGFR. CircZNF609 promotes the progression of LSCC via the miR-134-5p/EGFR axis, which might be the therapeutic target of LSCC.

## GRAPHICAL ABSTRACT

**Figure:**
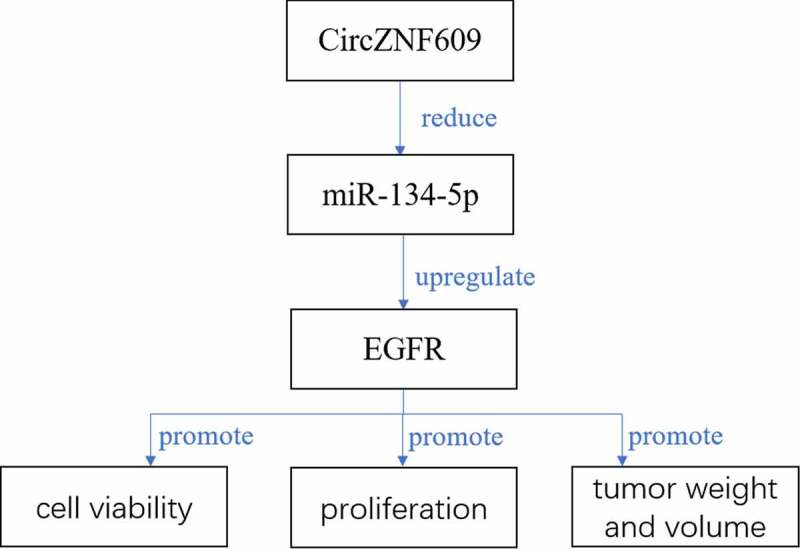

## Introduction

As one of the most common tumors of the head and neck [1,2], the incidence rate of laryngeal squamous cell carcinoma (LSCC) is the second highest in head and neck malignant tumors, and the eleventh in systemic tumors [3]. In recent years, the incidence of LSCC has significantly increased due to environmental pollution and smoke [4]. The treatment of early laryngeal cancer is mainly surgical resection [5]. In advanced laryngeal cancer, surgical treatment, radiotherapy (chemotherapy) or radioimmunotherapy are comprehensively selected according to the physical conditions, operative operability, and the presence or absence of metastasis of patients [6,7]. Active comprehensive treatment has greatly improved the prognosis of laryngeal cancer, but the overall survival rate of laryngeal cancer is still poor due to local recurrence and cervical lymph node metastasis [8]. Most patients eventually die from the recurrence and metastasis of cancer [9]. At present, the mechanisms of invasion and metastasis of malignant tumors are not fully understood. Therefore, it is of great importance to explore more valuable factors to improve the clinical outcome of LSCC.

Increasing number of biomarkers have been identified in the diagnosis or prognosis of patients with LSCC [10]. Studies have identified more than 3,000 circular RNAs (circRNAs), with only 1% been characterized [11,12]. CircRNAs are widely found in mammals and participate in gene regulation [13]. Studies have shown that circRNAs can regulate the progression of LSCC [14,15]. Xia *et al*. found that circRNA circ0067934 is upregulated in esophageal squamous cell carcinoma and promoted cancer cell proliferation [15]. CircZNF609 is abnormally expressed in many malignant tumor tissues [16]. Peng *et al*. reported that cir-ZNF609 is involved in the onset of Hirschsprung disease through the crosstalk with AKT3 by competing for miR-150-5p [16]. Li *et al*. showed that circ-ZNF609 depletion-repressed proliferation and cell cycle transition, and induced apoptosis of NPC cells by regulating the miR-188/ELF2 axis [17]. However, studies on the regulation of circZNF609 in LSCC are lacking.

In recent years, great progresses have been made in the study of microRNAs (miRNAs) [18]. MiRNAs exert their functions by specifically interacting with target genes to regulate their expression, leading to regulation of downstream signaling pathways as well as cell proliferation, fat metabolism, and various other biological processes [19,20]. Studies have identified several miRNAs that are abnormally expressed in LSCC tissues [21,22]. Previous studies have shown that 9 miRNAs were upregulated as the lesions become more malignant [23]. Cui *et al*. showed that the MIR155HG/miR‑155‑5p/SOX10 axis plays an important role in promoting the progression of LSCC and therefore may serve as a potential therapeutic target for LSCC treatment [24]. MiR-134-5p can inhibit the growth of gastric cancer, lung cancer and breast cancer [25]. Tong *et al*. found that the circZNF609/miR-134-5p/BTG-2 axis regulates the proliferation and migration of glioma cells [26]. Another study showed that long non-coding RNA (lncRNA) LUCAT1 promotes the proliferation and invasion of gastric cancer cells by regulating the miR-134-5p/YWHAZ axis [27]. However, the role of miR-134-5p in the development of LSCC is still elusive. Extensive studies have shown that circRNAs could exert their functions by regulating miRNAs and downstream target genes [28]. Epidermal growth factor receptor (EGFR) belongs to the ERBB protein family, most of which are membrane receptor tyrosine kinases that are activated by binding ligands. Overexpression of EGFR ligands and mutant formation are the main mechanisms leading to tumorigenesis [29]. One study reported that reciprocal regulation between ADAM17 and miR-145 results in aberrant activation of the EGFR signaling, suggesting that inhibition of ADAM17 might be an ideal therapeutic strategy for the treatment of GBM. In this study, we hypothesized that circRNA ZNF609 may regulate LSCC progression by regulating miR-134-5p. This study was carried out to explore the interactions among circRNA ZNF609, miR-134-5p, and EGFR in cervical cancer.

## Materials and Methods

### Patient information

A total of 42 LSCC samples and paired paracancerous tissue specimens were collected from the clinical sample bank of the Second Hospital of Hebei Medical University. Patients without chemotherapy or radiation therapy were selected. All patients signed the written consent. This study was approved by the Research Ethics Committee of the Second Hospital of Hebei Medical University.

### Cell culture and transfection

The human LSCC cell lines TU177, TU686, TU212, LSC-1 and Hep-2 were obtained from Shanghai Institute of Biological Science Cell Center. Hep-2 cells were cultured in F12K medium (Invitrogen), and the remaining cells were cultured in RPMI 1640 medium (Invitrogen).

Cell transfection was carried out when cells in culture reached 60–80% confluence using Lipofectamine® 2000. Small interfering RNAs (siRNAs) targeting circZNF609#1 (si-circZNF609#1, 5ʹ-GTCAAGTCTGAAAAGCAATGA-3ʹ), circZNF609#2 (5ʹ-TGCCCTAGTACTACCCTGCAT-3ʹ) and circZNF609#3 (5’-TTGACTGCATCGTAGCCAAAC-3’) and negative control (si-NC, 5’-UUCUCCGAACGUGUCACGUTT-3’) were purchased from Shanghai GenePharma Co., Ltd. The miR-134-5p mimetic, agomir and controls were purchased from Guangzhou RiboBio Co., Ltd. The transfection concentrations of oligonucleotides were as follows: si-NC, 40 nM; si- circZNF609, 40 nM; si-NC, 40 nM; si-EGFR, 40 nM; miR-134-5p mimetic, 50 nM; and miRNA control, agomir and miRNA control, 50 nM. Lipofectamine® 2000 Reagent and si-RNAs or miR-mimic were diluted with serum-free DMEM medium, mixed together and incubated at room temperature for 20 min. This solution was subsequently added to LSC-1 and Hep-2 cells for transfection at 37°C for 4–6 h in a humidified incubator containing 5% CO_2_.

### Quantitative reverse transcription polymerase chain reaction (qRT-PCR)

The specific experimental method of qRT-PCR was as previously described [30]. Total RNAs were extracted from cells using TRIzol reagent. After reverse transcription, qRT-PCR was performed using the ViiA^TM^ 7 real-time PCR system. GAPDH and U6 were used as the internal controls. The primer sequences were:

CircZNF609 (forward): 5’-CAATCTTCTTATGCGGCGG-3’;

CircZNF609 (reverse): 5’-GTACCGGCGCAGTCAGG-3’;

miR-134-5p (forward): 5’-CAGGGACTGAGGGCAATCGT-3’;

miR-134-5p (reverse): 5’-TTCATCGCGGTCGAGGGCGG-3’;

EGFR (forward): 5’-GAAAGTGCTTCGAAAGCGAC-3’;

EGFR (reverse): 5’-TCGCCGAAGTACTTGTGGC-3’;

GAPDH (forward): 5’-ATCCACGGGAGAGCGACAT-3’;

GAPDH (reverse): 5’-CAGCTGCTTGTAAAGTGGAC-3’;

U6 (forward): 5’-ACAGATCTGTCGGTGTGGCAC-3’;

U6 (reverse): 5’-GGCCCCGGATTATCCGACATTC-3’.

### Animal experiment

BALB/c nude mice (n = 18, male, 7-week-old) were used in this study. Hep-2 cell were divided into three groups: si-NC, si-circZNF609#1 or si-circZNF609#1 + miR-134-5p. Hep-2 cell were infected or co-infected with corresponding lentivirus. Then, infected Hep-2 cells (1 × 10^7^ cells per 0.1 mL) were injected into nude mice right flank. Nude mice were sacrificed with inhalation of carbon dioxide. The size of the tumors was measured once a week. This study was conducted in accordance with the National Institutes of Health Laboratory Animal Care and Use Guidelines [31].

### Cell proliferation measurement

CCK8 assay was conducted to evaluate cell proliferation. Transfected cells were seeded into 96-well plates. The absorbance values were determined at 450 nm using a microplate reader [31].

### Cell colony formation assay

In 6-well plates, transfected cells with 41,000 cells per well were inoculated. Cells were then immobilized with methanol and stained with crystal violet (0.1%). Finally, the colonies were imaged.

### BrdU incorporation assay

Transfected cells were seeded into 96-well plates at a density of 2,000 cells per well. Cell proliferation was analyzed using the BrdU Cell Proliferation Assay Kit at 48 h post-transfection.

### Transwell invasion assay

Cell invasion and metastasis were detected by Transwell assays. Matrigel (20 μg) was used to pre-coat the Transwell upper chamber basement membrane, in which cells were cultured overnight. PBS was used to wash the cells for 3 times, and then cells were fixed with formaldehyde (90%). Cells were then stained with crystal violet solution for 15 min. Then photographs were taken under an inverted microscope [31].

### Dual luciferase reporter gene assay

The wild type or mutant sequences for the 3’-untranslated region (3’-UTR) of circZNF609 and miR-134-5p were cloned into the pmirGLO vector. These reporter plasmids and miR-134-5p inhibitor or mimic were co-transfected into cells. Luciferase activity was detected using a dual luciferase assay system (Promega) after 48 h of transfection [31].

***Immunohistochemical*** (***IHC) stainin***g

Tumor tissues were fixed in formalin, deparaffinized in paraffin and heated after hydration. Next, 0.01 M sodium citrate buffer solution was used to pull the antigen down from sections at 95°C for 15 min. Then, the sections were incubated with rabbit anti-Ki67 at 4°C for 12 h, followed by incubation with Goat-anti-rabbit IgG-HRP (1:500, Boster, Wuhan, China) at room temperature for 1 h. The section staining was performed using hematoxylin and diaminobenzidine (DAB) solution. The expression of Ki67 was observed under a microscope (Olympus, Tokyo, Japan) with brown particles as positive expression signal [31].

### Western blot analysis

Total proteins were extracted from transfected cells. Protein concentrations were measured using the BCA Protein Assay Kit. Equal amount of protein samples (50 μg) were separated with SDS-PAGE, followed by blocking with nonfat milk. Proteins were transferred to PVDF membrane, which was then incubated with anti-EGFR antibody, anti-PCNA, anti-MMP-2 (1:500 dilution; Abcam, Cambridge, UK) and anti-GAPDH antibodies (1:1,000 dilution; Abcam, Cambridge, UK) at 4°C overnight. Then, the membrane was incubated with secondary antibody (1:5,000). Western blot analysis was carried out as described in literature [32].

### Statistical Method

SPSS19.0 software was used for data analyses. Data were shown as the mean ± standard deviation (SD). Multigroup data were compared by one-way ANOVA and LSD test. P < 0.05 indicated that the difference was significant.

## Results

### The expression of circZNF609 in LSCC

**Figure 1.**
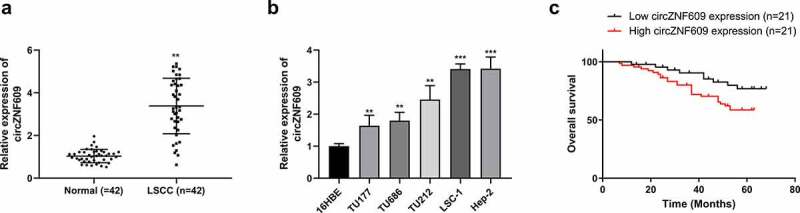
CircZNF609 was up-regulated in LSCC and predicts a poor prognosis for LSCC. (a) circZNF609 expression in LSCC tissues (n = 42). (b) circZNF609 expression in LSCC cell lines and human bronchial epithelial cells. (c) For analysis of LSCC patients’ survival, Kaplan-Meier analysis was used. ** P < 0.01, *** P < 0.001.

The expression of circZNF609 in laryngeal squamous cell carcinoma tissues were measured. The results showed that circZNF609 was upregulated in LSCC tissues compared to that in paired adjacent normal tissues (n = 42) (P < 0.01) (Figure 1(a)). In the LSCC cell lines (TU177, TU686, TU212, LSC-1 and Hep-2), the expression levels of circZNF609 were increased (P < 0.01) compared to that in human bronchial epithelial cells (16HBE) (Figure 1(b)).

With the median expression level of circZNF609 as the cutoff value, the 42 patients were divided into circZNF609 high- and low-expression groups. The circZNF609 high expression group exhibited lower overall survival rate than that in the circZNF609 low expression group (Figure 1(c), p < 0.01). The correlation analysis revealed that the expression of circZNF609 was tightly associated with tumor stage (P < 0.05). In contrast, drinking history, smoking history, age, gender, and clinical stage displayed no correlations with the expression of circZNF609 (Table 1). These results suggested that circZNF609 had potential LSCC carcinogenic effects.

**Table 1.** Correlations between circZNF609 and clinical characteristics of 42 laryngeal cancer patients

		circZNF609 level		
**Characteristics**	**n**	**High**	**Low**	***P* value**
Total case	42	21	21	
**Gender**				0.53
Male	26	14	12	
Female	16	7	9	
**Age (y)**				0.35
≤60	23	13	10	
>60	19	8	11	
**Smoking history**				0.47
Non‐smokers	10	4	6	
Current smokers	32	17	15	
**Drinking**				0.42
Drinker	34	18	16	
Non‐drinker	8	3	5	
**Tumor stage**				<0.05*
T1‐T2	22	16	6	
T3‐T4	20	5	15	
**Clinical stage**				0.53
I‐II	18	10	8	
III‐IV	24	11	13	

*p < 0.05

### Knockdown of circZNF609 inhibited LSCC growth in vitro

**Figure 2.**
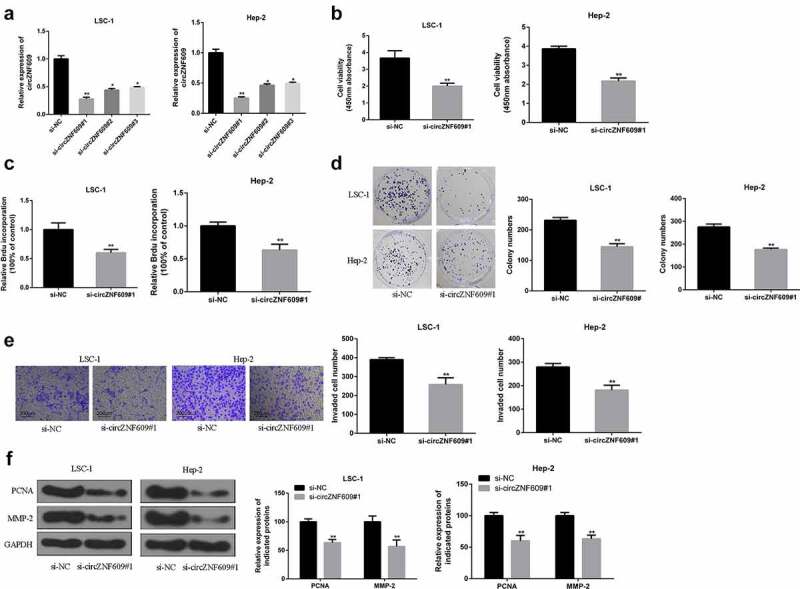
CircZNF609 promoted the malignant behavior of LSCC cells. (a) qRT-PCR was used to detect circZNF609#1, circZNF609#2, and circZNF609#3 transfection efficiency in LSCC cells. (b) cell viability analysis. (c) cell proliferation analysis (d) cell colony formation analysis. (e) cell invasion analysis (f) PCNA and MMP-2 protein expression.* p < 0.05, ** p < 0.01.

To explore the function of circZNF609 in LSCC, siRNAs were used to knockdown the expression of circZNF609. By transfecting three circZNF609 siRNAs (circZNF609#1, circZNF609#2, circZNF609#3), we found that circZNF609#1 could significantly downregulate the expression of circZNF609 in LSC-1 and Hep-2 cells (Figure 2(a)). LSC-1 and Hep-2 cell viability (Figure 2(b), p < 0.01) and proliferation rate (Figure 2(c), p < 0.01) were both significantly decreased in circZNF609-suppressed group. In addition, to further evaluate the ability of cell proliferation, cell colony formation assay was performed. In the circZNF609-silencing group, LSC-1 and Hep-2 cell colony formation was also reduced (Figure 2(d), p < 0.01). Besides, cell invasion ability was detected by transwell invasion assay. The number of cells in the circZNF609-silencing group was significantly reduced (Figure 2(e), p < 0.01). Moreover, in LSC-1 and Hep-2 cells, the expression levels of PCNA and MMP-2 in the circZNF609-suppressed group were significantly reduced (P < 0.01) (Figure 2(f)). These results suggested that circZNF609 promoted LSCC cell growth and metastasis.

### CircZNF609 served as a sponge of miR-134-5p

**Figure 3.**
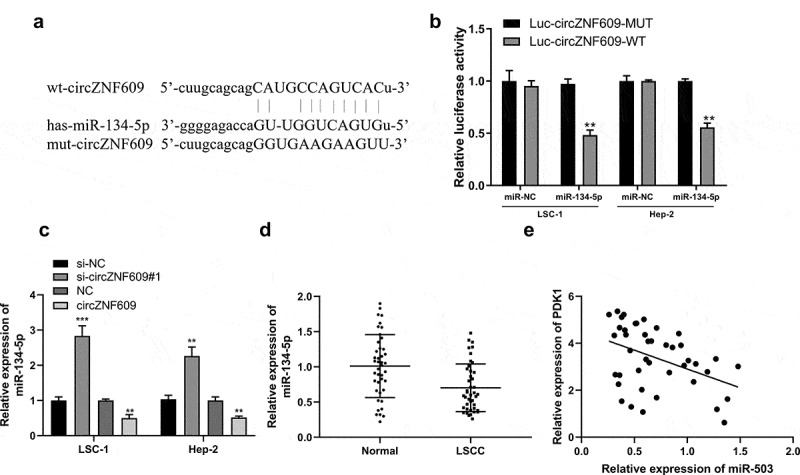
miR-134-5p is a circZNF609 target. (a) The binding site between circZNF609 and miR-134-5p (b) LSC-1 and Hep-2 cell luciferase activity analysis. (c) miR-134-5p expression in LSC-1 and Hep-2 cells. (d) miR-134-5p expression in adjacent normal and LSCC tissues (n = 42). (e) The correlation analysis in LSCC tissues (n = 42) (r = −0.662, P < 0.001). ** P < 0.01, *** P < 0.001.

Potential base pairs formed between circZNF609 and miR-134-5p were predicted by IntaRNA 2.0 [33]. Bioinformatics analysis showed that miR-134-5p was a potential target of circZNF609 (Figure 3(a)). Overexpression of miR-134-5p reduced luciferase activity of WT-circZNF609, but not the luciferase activity of mut-circZNF609 (Figure 3(b)). Knockdown of circZNF609 significantly increased the expression levels of miR-134-5p in LSC-1 and Hep-2 cells, but overexpression of circZNF609 reduced the expression levels of miR-134-5p (P < 0.01) (Figure 3(c)). Furthermore, the expression levels of miR-134-5p were reduced in LSCC tissues (P < 0.01) (Figure 3(d)) and correlated with the expression of circZNF609 in LSCC tissues (r = −0.662, P < 0.001) (Figure 3(e)). These results suggested that circZNF609 sponged miR-134-5p to exert its biological functions.

### CircZNF609 sponged and sequestered miR-134-5p to upregulate EGFR

**Figure 4.**
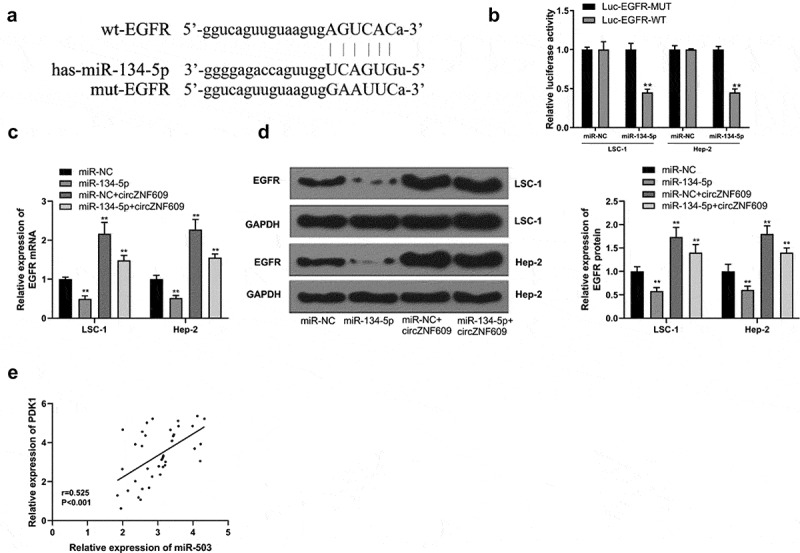
circZNF609 upregulated EGFR by sponging miR-134-5p. (a) Binding sites for EGFR 3’-UTR and miR-134-5p. (b) Luciferase activity analysis. (c) EGFR mRNA expression. (d) EGFR protein expression. (e) The correlation analysis in LSCC tissues (n = 40). #vs miR-134-5p simulation group, * vs control group, ** vs control, P < 0.01, vs miR-NC + circZNF609, #P < 0.05.

Next, potential base pairs formed between EGFR and miR-134-5p were predicted by IntaRNA 2.0 [33]. Bioinformatics analysis also identified that EGFR was a target of miR-134-5p (Figure 4(a)). Ectopic expression of EGFR inhibited the luciferase activity of WT-miR-134-5p, but not the luciferase activity of mut-miR-134-5p (Figure 4(b)). Overexpression of miR-134-5p reduced the expression levels of EGFR, and overexpression of circZNF609 increased the expression levels of EGFR, while co-transfection of circZNF609 + miR-134-5p reversed circZNF609 and miR-134-5p mediated EGFR expression (Figures 4(c,d)). Furthermore, the expression of circZNF609 was positively correlated with EGFR in LSCC tissues (r = 0.525, P < 0.001) (Figure 4(e)). These results suggested that circZNF609 enhanced the expression of EGFR by sponging miR-134-5p.

### circZNF609 promoted LSCC progression by inhibiting the miR-134-5p/EGFR axis

**Figure 5.**
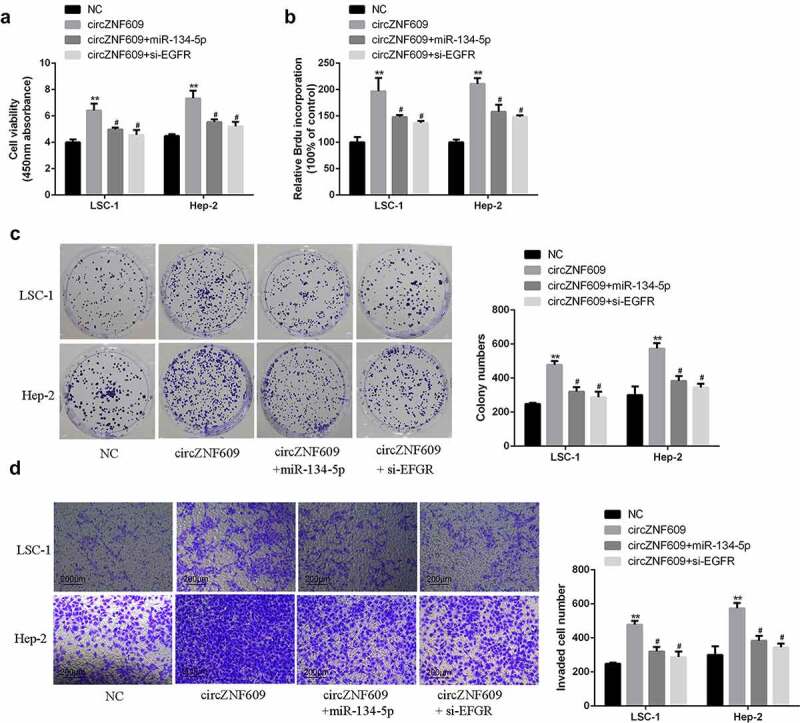
(a) Cell viability analysis. (b) Cell proliferation analysis. (c) Cell colony formation analysis. (d) Cell invasion analysis. #vs circZNF609 group, * vs control vector group, **P < 0.01, #P < 0.05 compared with circZNF609.

To investigate cell viability and proliferation, CCK-8 assay, BrdU incorporation assay and cell colony formation assay were performed. The results showed that overexpression of circZNF609 significantly increased LSC-1 and Hep-2 cell viability and proliferation rate (Figures 5(a-c)), which was reversed by overexpression of miR-134-5p or knockdown of EGFR. In transwell invasion assay, overexpression of circZNF609 increased cell invasion, which was reversed by either overexpression of circZNF609 and miR-134-5p or silencing of EGFR (Figure 5(d)). These results demonstrate that EGFR and miR-134-5p mediated the function of circZNF609-in LSCC cells.

### The miR-134-5p/EGFR axis mediated the function of circZNF609 in LSCC progression

**Figure 6.**
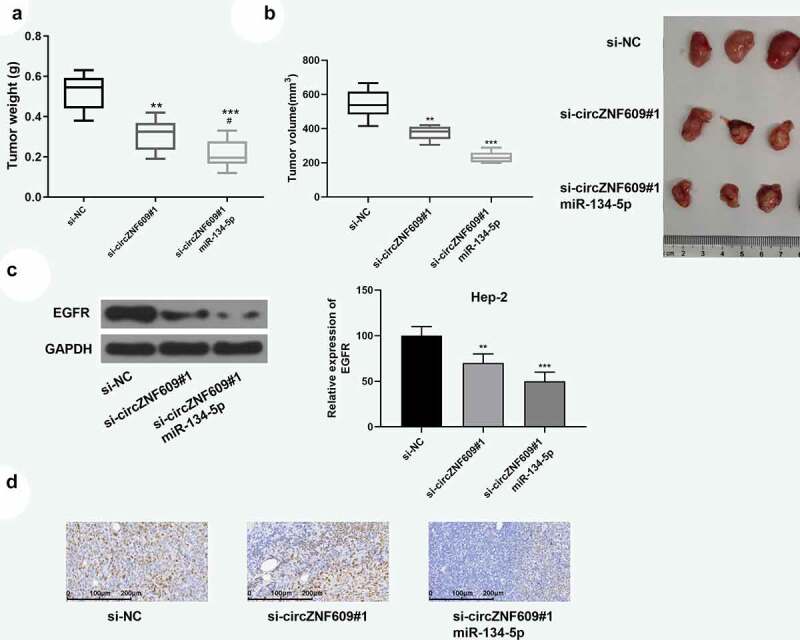
circZNF609 modulated the miR-134-5p/EGFR axis to promote LSCC progression in vivo. Hep-2 cells (1 × 10^7^ cells per 0.1 mL) stably transfected with si- circZNF609#1 or si-circZNF609#1+ miR-134-5p, si-NC (n = 6). Hep-2 cells were implanted subcutaneously into nude mice, and tumor weight (a) and volume (b) was evaluated. (c) EGFR protein expression. (d) IHC staining was used to detect ki-67 expression (×100, scale = 100 μm). vs si-circZNF609, #P < 0.05, *** P < 0.001, ** P < 0.01, vs p-NC.

Previous experiments have confirmed that circZNF609 induced promotion of LSCC progression through inhibiting the miR-134-5p/EGFR axis. Next, the effect of overexpression of miR-134-5p on tumor promotion induced by circZNF609 was verified. The function of circZNF609 in LSCC progression was further explored *in vivo* by establishing a xenograft tumor model by subcutaneous injection of Hep-2 cells (n = 6 per group). On the 9^th^ day, si-circZNF609#1, miR-134-5p agomir or a negative control was injected into the mouse tumor. Knockdown of circZNF609 reduced tumor weight and volume, which was reversed by overexpression of miR-134-5p (Figure 6(a,b)). In addition, as shown in Figure 6(c), knockdown of circZNF609 could inhibit the expression of EGFR, which was reversed by overexpression of miR-134-5p. In addition, knockdown of circZNF609 significantly reduced the expression levels of Ki67, and overexpression of miR-134-5p further reduced the expression levels of Ki67 (Figure 6(d)). These data indicated that the miR-134-5p/EGFR axis mediated circZNF609 function in LSCC progression.

## Discussion

LSCC is a malignant tumor that occurs in the epithelium of the laryngeal mucosa [34]. It is one of the most common malignant tumors in 50–60 years-old male [35]. However, the pathogenesis of LSCC is still unclear. It is speculated that the major risk factors for LSCC are a combination of factors such as smoking, drinking, air pollution, and viral infection [36,37]. Extensive studies have confirmed that LSCC requires lifelong treatment and monitoring [38]. Therefore, the diagnosis and evaluation of LSCC in the early stages, and a better understanding of its biological characteristics will be the key to future LSCC prevention.

Molecular marker testing helps physicians to develop more individualized treatment plans for LSCC patients [39]. CircRNAs play critical roles in LSCC and are promising biomarkers for the diagnosis and progression of LSCC [40]. It was reported that circZNF609 sponged miR-615 to regulate retinal neurodegeneration [41]. In breast cancer, circZNF609 sponges miR-145-5p to promote cancer cell invasion, migration, and growth by upregulating p70S6K1 [42]. Another study reported that circZNF609 regulates glioma cell migration and proliferation via the miR-134-5p/BTG-2 axis [26]. In nasopharyngeal carcinoma, circZNF609 sponges miR-338-3p to promote cell glycolysis, invasion, migration, and proliferation by regulating HRAS [43]. However, how circZNF609 promotes carcinogenesis in LSCC remains elusive. Our study showed that the expression levels of circZNF609 increased in LSCC, and overexpression of circZNF609 reduced patient survival rate. MMP-2 can degrade collagen indirectly, which can be used to study the migration of cancer cells after labeling. PCNA can reflect the proliferation of cells and is an important index to evaluate the proliferation status and malignant potential of cells. Silencing of circZNF609 inhibited cell proliferation and reduced cell invasiveness, and significantly reduced the expression levels of PCNA and MMP-2 protein. *In vivo* experiments showed that the tumor volume and weight in the circZNF609-silence group were significantly reduced, indicating that the development of LSCC could be inhibited by supressing the expression of circZNF609.

miRNAs are the most widely studied class of lncRNAs and can function as the target of circRNA to regulate cell apoptosis, differentiation and proliferation by inhibiting or degrading translation of target mRNAs [44]. It was shown that miR-134-5p was involved in tumor development and inhibits tumor cell invasion and migration [45]. Here, miR-134-5p was identified to be a target of circZNF609, and its expression was regulated by circZNF609. Overexpression or knockdown of circZNF609 altered the expression of miR-134-5p. MiR-134-5p was downregulated and negatively correlated with the expression of miR-134-5p in LSCC tissues.

EGRF belongs to the Erb B family of type I tyrosine kinases and is an important transmembrane receptor [46]. The binding of EGFR gene with the EGF or TGF-α receptors initiates a series of downstream cascade reactions, which eventually leads to the increase of gene transcription levels in the nucleus and the proliferation and transformation of cells [47]. Studies have shown that miR-134-5p significantly inhibited the expression of EGFR in cancer cells [48]. Here, EGRF was a target of miR-134-5p and regulated by miR-134-5p. Overexpression of circZNF609 increased the expression levels of EGRF, which was reversed by co-transfection of circZNF609 + miR-134-5p. Moreover, circZNF609 positively correlated with EGRF in LSCC tissue. In addition, overexpression of miR-134-5p and silencing of EGRF reversed circZNF609 mediated LSCC cell invasion and proliferation, suggesting that circZNF609 upregulated EGRF by regulating miR-134-5p. However, there are some limitations in this study, for example, the expression of circZNF609 in the tumor section was not detected by Immunohistochemical staining. The dose-dependent intervention of circZNF609 in the cell culture level was not investigated. We will improve these limitations in our future studies.

## Conclusion

CircZNF609 promoted LSCC invasion and proliferation by regulating miR-134-5p, thus activating EGRF, indicating that circZNF609 may be an oncogene in LSCC. Our findings will provide experimental basis for clinical prognosis judgment and targeted intervention therapy for LSCC.

## Supplementary Material

Supplemental Material

## Data Availability

The data that support the findings of this study are available from the corresponding author upon reasonable request.

## List of abbreviations

Laryngeal squamous cell carcinoma (LSCC)Circular RNA (circRNA)
