# Supplementary figures and images for: Circular RNA ZNF609 promotes laryngeal squamous cell carcinoma progression by upregulating epidermal growth factor receptor via sponging microRNA-134-5p
Source: Bioengineered. 2022 Mar 2;13(3):6929–41. doi: 10.1080/21655979.2022.2034703 (PMC8973624; doi:10.1080/21655979.2022.2034703)

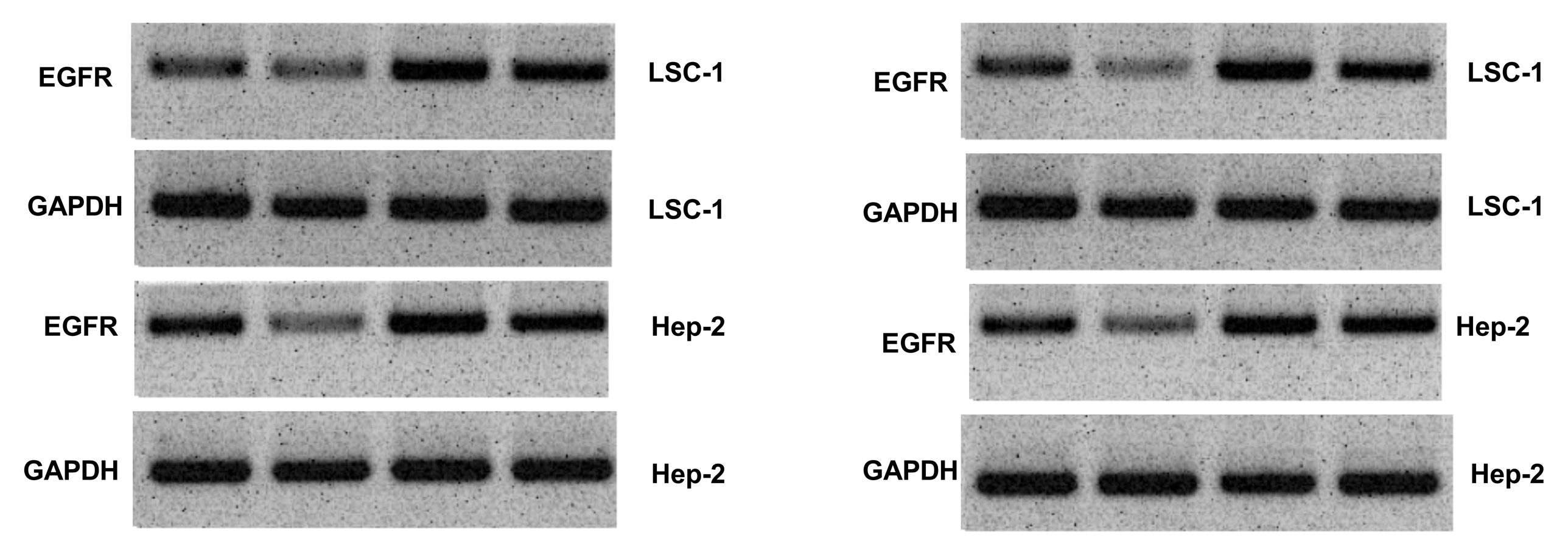

Supplement: Supplemental Material [file KBIE_A_2034703_SM8379.tif]
